# Major Ethnic Populations Are Significantly Differentiated at the Glioblastoma Multiforme Candidate Loci

**DOI:** 10.3390/ijms27104424

**Published:** 2026-05-15

**Authors:** Volodymyr Mavrych, Maryam Alamil, Olena Bolgova, Volodymyr Dvornyk

**Affiliations:** 1 College of Medicine, Alfaisal University, Riyadh 11533, Saudi Arabia; vmavrych@alfaisal.edu (V.M.); obolgova@alfaisal.edu (O.B.); 2 College of Science and General Studies, Alfaisal University, Riyadh 11533, Saudi Arabia; malamil@alfaisal.edu

**Keywords:** glioblastoma multiforme, population genetics, polygenic risk score, ethnic disparities, GWAS, risk loci

## Abstract

Glioblastoma multiforme (GBM) is the most aggressive primary brain tumor, with well-documented incidence disparities across ethnic populations: highest in Europeans and lowest in East Asians and Africans. Still, the genetic basis of these differences remains poorly understood. This study assessed whether population-level differences in GBM risk allele frequencies correlate with ethnic disparities in prevalence. We analyzed 673 genome-wide significant GBM candidate loci across five ethnic superpopulations and 26 subpopulations using phased genotype data from the 1000 Genomes Project Phase 3. Population genetic structure was characterized using allele frequencies, heterozygosity, Wright’s fixation index, analysis of molecular variance (AMOVA), Nei’s genetic distances, and principal coordinate analysis. Risk allele enrichment was visualized via hypergeometric heatmaps, and polygenic risk scores were compared using Kruskal–Wallis and Dunn’s tests. Significant interpopulation differentiation was detected across all superpopulation pairs (*p* < 0.001). European populations had the highest polygenic risk scores, followed by South Asian and Admixed American populations, while East Asians had the lowest. Allele frequencies at key loci, including rs634537 (*CDKN2B-AS1*) and rs55705857 (*CCDC26*), differed up to tenfold. Finnish populations showed an elevated risk consistent with founder effects. Population genetic structure at GBM risk loci correlates with ethnic incidence disparities, underscoring the need for ancestry-specific approaches in risk modeling and trans-ancestry studies.

## Simple Summary

Brain cancer rates differ markedly around the world, with people of European descent developing glioblastoma (the deadliest form of brain cancer) far more often than those of African or East Asian descent. While lifestyle and environmental factors play a role, inherited genetic differences between populations may also contribute to these disparities. Every person carries genetic variants that may slightly increase or decrease disease risk, and the frequencies of these variants differ across populations. We examined nearly 700 genetic variants associated with glioblastoma risk and compared their frequencies across five major ethnic groups worldwide. European populations carry the highest concentration of risk variants, while East Asian populations carry the fewest, a pattern that mirrors real-world differences in brain cancer rates. These findings suggest that genetic makeup contributes to ethnic disparities in glioblastoma and highlight the need to include diverse populations in future research to improve cancer risk prediction.

## 1. Introduction

Glioblastoma Multiforme represents the most common and lethal primary malignant brain tumor in adults, accounting for approximately 54% of gliomas, with a mean survival of only 15 months [1]. It is characterized by rapid proliferation, extensive infiltration into surrounding brain tissue, and a high degree of molecular and cellular heterogeneity, which collectively contribute to its dismal prognosis and resistance to conventional therapies [2]. The pathophysiology of GBM is driven by a complex interplay of genetic and epigenetic alterations that disrupt key cellular pathways, including those controlling cell cycle progression, apoptosis, and DNA repair. These tumors can arise de novo as primary GBM, which constitutes the vast majority of cases (approximately 90–95%), or they can progress from lower-grade diffuse astrocytomas (Grade II or III) in a process termed secondary GBM [3]. While histologically indistinguishable, primary and secondary GBMs are thought to evolve through distinct genetic pathways, affecting patients at different ages and exhibiting different molecular profiles [4].

The genetic landscape of GBM is highly complex, with several key driver alterations identified. The most common genetic events include mutations in the *TERT* promoter, which lead to reactivation of telomerase and cellular immortality, and are found in a significant proportion of GBMs [5]. Other frequent alterations include amplification of the *EGFR* gene, loss of the tumor suppressor *PTEN* (through deletion or inactivating mutation), *TP53* mutations, and homozygous deletions of the *CDKN2A* locus [6]. The *IDH1* and *IDH2* genes are also critical in glioma classification; while mutations in these genes are rare in primary GBM, they are a hallmark of secondary GBM and lower-grade gliomas and are associated with a significantly better prognosis [7]. The 2021 WHO classification of central nervous system tumors has further refined the definition of GBM, now termed “Glioblastoma, IDH-wildtype,” which requires the presence of microvascular proliferation or necrosis, or at least one of three genetic alterations: *TERT* promoter mutation, *EGFR* gene amplification, or +7/−10 chromosome copy number changes [8]. This molecular-based classification underscores the importance of integrating genetic information into the diagnosis and management of GBM, paving the way for more personalized therapeutic strategies.

The epidemiology of GBM exhibits significant variations across populations. In the United States, the age-adjusted incidence rate of GBM is approximately 3.19 per 100,000 persons, with a median age at diagnosis of 64 years [9]. The incidence is notably higher in males than in females, with a male-to-female ratio of about 1.6:1 [10]. Studies of U.S. cohorts have consistently shown that the incidence of GBM is highest among individuals of European (EUR) ancestry, followed by those of Admixed American (AMR) descent, while it is significantly lower in populations of African (AFR) and East Asian (EAS) ancestry [11,12]. For instance, the incidence rate in European Americans is reported to be 2.5 times higher than in African Americans [11,13]. These disparities are not fully understood but are likely influenced by a combination of genetic predisposition and environmental exposures.

The problem of between-ethnic disparities in disease prevalence is well-known and has been extensively studied (e.g., [14,15,16]). Interethnic differences in population genetic structure were implicated as one of the primary factors contributing to the above disparities [17,18,19,20,21].

In this study, we analyzed the genetic structure of ethnic populations worldwide at GBM risk loci using data from the 1000 Genomes Project [22] and tested whether differences in population genetic structure might correlate with the reported disease prevalence across ethnicities.

## 2. Results

### 2.1. Allelic Structure of the Populations

Quite a significant number of the analyzed loci were monomorphic in at least one ethnic superpopulation. Specifically, 268 loci had fixed alleles in AFR, 68 in AMR, 511 in EAS, 11 in EUR, and 156 in SAS. Alleles at 105 loci were fixed in three populations: AFR, EAS, and SAS; 27 loci had fixed alleles in all populations except EUR. None was fixed in all populations. A relatively small number of loci showed a significant departure from HWE, with the largest number (52) in EUR and the smallest (13) in AFR (Table S2).

**Figure 1:**
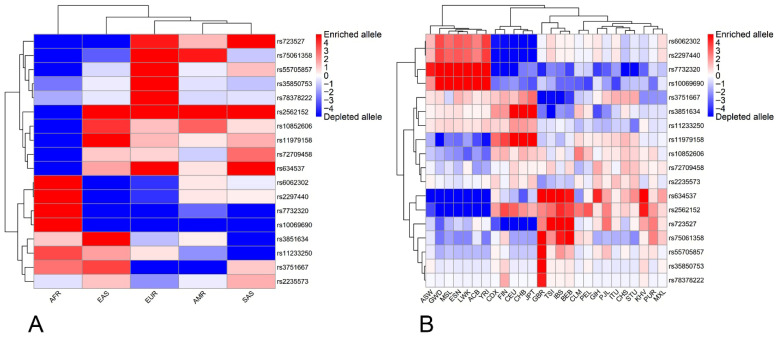
A heatmap of the effect allele frequencies in the studied ethnic populations. The color scale represents the depletion (blue) and enrichment (red) of the allele as compared to the average allele frequency in the whole sample (white). The scale numbers indicate −log_10_ values. (**A**)—ethnic superpopulations; (**B**)—ethnic subpopulations. For the population designations, see Table 1.

The heatmaps depicting enrichment and depletion of effect allele frequencies are presented in Figure 1. A significant proportion of loci exhibited markedly different allele frequency patterns across the ethnic superpopulations (Table S3). The most significant differences were observed between the EUR and AFR superpopulations: about half of the loci were depleted in the former and enriched in the latter, and vice versa (Figure 1A). Hierarchical clustering of the five superpopulations revealed a primary divergence between the African (AFR) superpopulation and other continental groups. Smaller blocks of loci show specific enrichment in the EUR and SAS superpopulations, suggesting that the genetic risk profile for GBM may be driven by distinct sets of variants depending on ancestry. The SAS and AMR superpopulations exhibited intermediate-frequency patterns, reflecting their complex demographic histories and potential admixture.

The analysis of 26 subpopulations (Figure 1B) showed significant heterogeneity in the observed allele frequency patterns. The most consistent profiles were observed only among African subpopulations, which formed a highly cohesive cluster with consistent loci enrichment patterns.

Frequencies of effect alleles at some loci differed quite significantly across the populations. For example, allele rs634537-G, an intronic variant of the *CDKN2B-AS1* gene (*CDKN2B* and *CDKN2A* antisense cis and trans regulatory RNA 1), has a ten-fold lower frequency in AFR than in any other superpopulation. Similar differences in magnitude were observed for effect alleles at several other risk loci (e.g., rs75061358, rs35850753, rs55705857; see Table S4a).

Significant variation in effect allele frequencies was also observed among subpopulations of the same ethnicity. For example, alleles rs75061358-G, rs35850753-T, rs78378222-G and the others (Table S4b).

Notably, some effect alleles were not detected in either some subpopulations or superpopulations. For example, allele rs78378222-G was absent in two superpopulations (AFR and AMR); in the other two (EAS and SAS), it was found only in one subpopulation of each (Table S4).

### 2.2. Intra- and Interpopulation Genetic Diversity

The diversity parameters of the five superpopulations are given in Table 1. Overall, the level of genetic diversity at the GBM candidate loci was quite low. Among the superpopulations, the highest and lowest levels of diversity were observed in EUR and EAS, respectively. Subpopulations of the same ethnicity showed similar levels of diversity; the greatest variation was observed among American subpopulations. All populations were characterized by very low levels of inbreeding (Table S5).

Despite low genetic diversity, the populations were significantly differentiated at the analyzed loci (Table 2 and Table S6). This differentiation is visualized in the Principal Coordinate Analysis (PCoA) plot.

**Figure 2:**
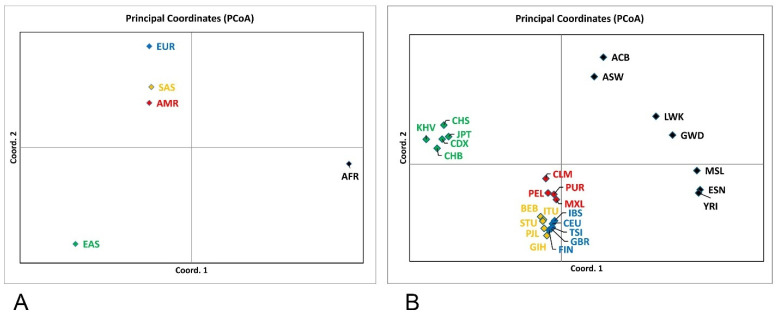
Principal Coordinate Analysis of the ethnic super- and subpopulations. (**A**)—ethnic superpopulations; (**B**)—ethnic subpopulations. The superpopulations and respective subpopulations are color-matched. For the population designations, see Table 1.

The PCoA grouped the ethnic subpopulations into well-defined clusters corresponding to the five superpopulations (Figure 2). The analysis revealed clear patterns of genetic structure among the five continental superpopulations. The AFR cluster occupied a distinct position along the primary coordinate axis, indicating the greatest degree of genetic differentiation relative to all other groups. In contrast, the EAS superpopulation formed a separate, well-defined cluster along the opposite direction of the secondary coordinate, reflecting substantial divergence from both AFR and EUR superpopulations. The EUR, SAS, and AMR superpopulations clustered closely together, indicating a high degree of similarity in allele-frequency patterns, consistent with shared West Eurasian ancestry components. The intermediate placement of AMR between EUR and SAS suggests the influence of multiple ancestral contributions typical of admixed American populations. Overall, the PCoA recapitulates known global population structure, with African divergence dominating the primary axis and East Asian differentiation contributing prominently to the secondary axis. At the same time, EUR and AMR superpopulations remain comparatively proximate in multidimensional genetic space. Interestingly, EAS and AFR, which were the most distinct from the others, also possessed the lowest genetic diversity (Table 1).

At the subpopulation level, the overall structure recapitulates the superpopulation pattern while revealing within-continent heterogeneity. African groups (ESN, MSL, YRI, GWD) cluster together, thus confirming the strongest differentiation from non-African populations. The diaspora populations ASW and ACB are clearly displaced toward AMR/EUR, consistent with the known admixture of West Eurasian and American components. East Asian subpopulations form a compact cluster, indicating minimum differentiation among them at the loci considered. European subpopulations cluster compactly, with FIN slightly offset from the others, reflecting subtle intra-European structure. American and South Asian subpopulations occupy intermediate positions between EUR and EAS/AFR, demonstrating varying admixture proportions characteristic of these groups.

Further strong support for the significant interpopulation differentiation comes from the results of the pairwise exact *G*-test for genic and genotypic differentiation [23]. The analysis across all 673 loci yielded highly significant differentiation (*p* << 10^−4^) between pairs of superpopulations. Likewise, such a high degree of differentiation was observed between subpopulations of different ethnicities. Most subpopulations of the same ethnic ancestry were not significantly differentiated, except CDX and CHB (Table S7).

### 2.3. Composite Genetic Scores at the GBM Risk Loci

**Figure 3:**
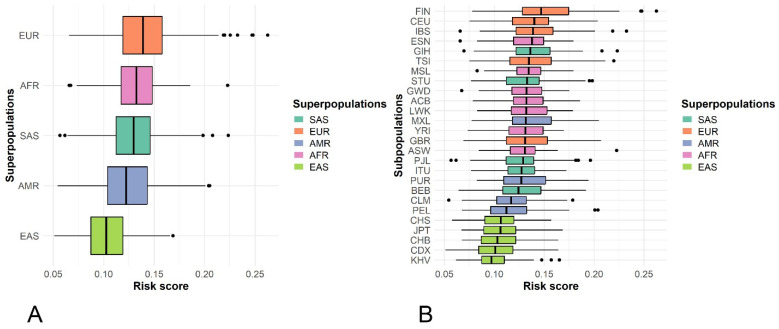
Box plot diagram of population-specific composite genetic risk scores for GBM: (**A**)—ethnic superpopulations; (**B**)—ethnic subpopulations. The center line of the box plot represents the median, the edges of the box indicate the 25th and 75th percentiles; the whiskers extend 1.5 times the interquartile range from these percentiles, and circles denote outliers. For the population designations, see Table 3.

There is a clear gradient of genetic risk across ethnic backgrounds. The EUR superpopulation exhibits the highest median population-specific polygenic risk scores (psPRS), followed by AFR and SAS. In contrast, EAS has the lowest median risk, suggesting a significantly different load of risk-associated alleles compared to Western cohorts. The mean unweighted composite genetic risk score for GBM across all studied populations was 0.127 ± 0.029, the lowest in East Asians (0.104 ± 0.022) and the highest in Europeans (0.140 ± 0.031; Figure 3A).

The analysis at the subpopulation level revealed significant intracontinental variation (Figure 3B). Within the EUR cluster, the Finnish (FIN) population shows the highest overall risk and the greatest number of high-risk outliers. This may be attributed to historical founder effects and genetic isolation, which are known to concentrate on specific risk variants. The AMR subpopulations (e.g., MXL and PUR) display broad risk ranges, apparently due to the varying effects of European, African, and Indigenous ancestry on individual risk profiles. Both the EAS and AFR subpopulations show the most compact risk distributions and lower medians, indicating a potentially lower prevalence of the specific GBM risk alleles.

There was no significant difference in psPRS between males and females.

It is worth noting that the unweighted psPRS is a significant underestimate of the true (weighted) psPRS because it does not account for the effect size of the risk allele. For example, the unweighted psPRS for the EUR superpopulation was 0.140 ± 0.031, about fourfold smaller than the weighted psPRS (0.688 ± 0.127).

**Table 3:** Populations analyzed in the present study.

Code	Size	Description
ALL	2504	All phase 3 individuals
**AFR**	**661**	**African**
ACB	96	African Caribbean in Barbados
ASW	61	African Ancestry in Southwest US
ESN	99	Esan in Nigeria
GWD	113	Gambian in Western Division, The Gambia
LWK	99	Luhya in Webuye, Kenya
MSL	85	Mende in Sierra Leone
YRI	108	Yoruba in Ibadan, Nigeria
**AMR**	**347**	**American**
CLM	94	Colombian in Medellin, Colombia
MXL	64	Mexican Ancestry in Los Angeles, California
PEL	85	Peruvian in Lima, Peru
PUR	104	Puerto Rican in Puerto Rico
**EUR**	**503**	**European**
CEU	99	Utah residents with Northern and Western European ancestry
FIN	99	Finnish in Finland
GBR	91	British in England and Scotland
IBS	107	Iberian populations in Spain
TSI	107	Toscani in Italy
**EAS**	**504**	**East Asian**
CDX	93	Chinese Dai in Xishuangbanna, China
CHB	103	Han Chinese in Beijing, China
CHS	105	Southern Han Chinese, China
JPT	104	Japanese in Tokyo, Japan
KHV	99	Kinh in Ho Chi Minh City, Vietnam
**SAS**	**489**	**South Asian**
BEB	86	Bengali in Bangladesh
GIH	103	Gujarati Indian in Houston, TX
ITU	102	Indian Telugu in the UK
PJL	96	Punjabi in Lahore, Pakistan
STU	102	Sri Lankan Tamil in the UK

The pairwise comparison of the unweighted psPRS across the superpopulations using Dunn’s test showed that all pairs differed significantly (Table 4). Within-ethnic differences in psPRS were much less pronounced (Table S8).

## 3. Discussion

Glioblastoma is overwhelmingly a sporadic cancer, although a measurable inherited (germline) component does exist. Familial predisposition and rare Mendelian syndromes account for roughly 5% and 1–2% of GBM cases, respectively [24]. GBM apparently does not have a strong genetic component. Twin-study–based heritability estimates are not available for GBM specifically, but large epidemiological and human-genetic analyses place the heritability of glioma (the tumor class that includes GBM) in the moderate range, typically interpreted as approximately 20–30% for glioma overall and 26% for GBM specifically [25]. A recent sibling-based study of nervous tumors estimated their heritability to be in the same range, i.e., 29% (95% CI, 19–39%) [26]. These estimates indicate that while most cases occur sporadically, common SNP variants account for about a quarter of the disease’s variance. While *h*^2^ is 26%, the specific risk loci identified through genome-wide association studies (GWAS)—such as *TERT*, *EGFR*, and *RTEL1*—collectively explain only about 1.3% of the total variance. This massive gap between 26% and 1.3% indicates that GBM is highly polygenic, meaning it is influenced by thousands of variants whose effects are too small to be detected individually in current studies.

According to the common disease–common variant hypothesis, genetic susceptibility to common multifactorial disorders is driven primarily by common alleles, i.e., those with a population frequency ≥ 5% [27,28]. This expectation, however, contradicts the well-documented ethnic differences in the prevalence of many complex diseases [29,30,31,32,33,34]. Although such disparities have traditionally been attributed to socioeconomic and environmental factors [35,36], a growing body of evidence suggests that ancestry-related differences in population genetic structure may also be a significant factor [17,18,19,37]. Recent studies have reported that variation in GBM risk allele frequencies accounts for >20% reduction in disease risk in Africans vs. Europeans and >50% risk decrease in East Asians vs. Europeans, indicating a meaningful genetic contribution to differences in prevalence [38,39].

### 3.1. Locus-Specific Contributors to Ancestry-Dependent Risk

Several GBM/glioma risk loci exhibit order-of-magnitude differences in risk allele frequencies (RAF) across populations, creating a natural substrate for interpopulation variation in polygenic liability. The 8q24.21 (*CCDC26*) locus—specifically rs55705857-G—confers an exceptionally large effect for *IDH* mutant glioma (OR ≈ 5–6) and is now supported by causal mechanistic evidence showing enhancer disruption and *MYC* dysregulation [40]; importantly, the RAF of rs55705857 varies substantially across ancestries, being the highest in Europeans. Beyond continental structure, founder effects and demographic history can concentrate risk variants within subpopulations—Finns are a classic example. The Finnish population history (bottlenecks, drift, and regional substructure) (e.g., [41]) showed enrichment of low-frequency deleterious alleles, with practical implications for disease genetics and risk distribution. Our finding that FIN deviates notably from other European subpopulations—and harbors more outliers in the risk score distribution—fits established population genetic expectations (Figure 3B).

In parallel, the 9p21.3 (*CDKN2B AS1/ANRIL*) region (rs634537-G) has repeatedly been associated with glioma susceptibility—including in children and across ancestries—and functional enhancer activity affecting *CDKN2B AS1* has been demonstrated [34]. Our observation that many of these loci are fixed or nearly fixed within certain superpopulations mirrors the reported RAF heterogeneity in these and related regions, suggesting plausible locus-specific channels through which population structure influences aggregate GBM risk.

Notably, early high-grade glioma GWAS identified variants at *CDKN2B* and *RTEL1* loci with different frequency profiles across continents; together with newer findings at telomere maintenance genes (*TERT*, *OBFC1/STN1*), these patterns reinforce a biological axis (cell cycle/telomere control) through which ancestry modulated RAFs can reshape risk at the population level.

Across the five continental superpopulations, we observed extensive between-groups heterogeneity in allele frequencies at GBM candidate loci, with many sites monomorphic in at least one superpopulation. The number of monomorphic loci differs significantly among superpopulations, from 511 in EAS to only 11 in EUR. This indicates that European populations maintain substantially greater genetic variation at GBM risk loci. Consistent with this pattern, only a small subset of loci has departed from the Hardy–Weinberg equilibrium within populations, and the number of HWE departures was modest overall (maximum of 52 in EUR), arguing against pervasive genotyping error or strong within-population selection as global drivers of the observed structure. Together, these results indicate that the allelic landscape of GBM-associated variants is not uniformly polymorphic worldwide and that many loci are effectively uninformative within certain ancestries.

### 3.2. Enrichment/Depletion Patterns and Hierarchical Clustering

Heatmap analysis reinforced these conclusions by revealing pronounced enrichment/depletion blocks that segregate by ancestry. The sharpest contrast occurred between EUR and AFR, with many loci showing relative depletion in EUR and enrichment in AFR. Importantly, the enrichment/depletion metric is defined relative to the global mean at each locus; therefore, a “depleted” signal (e.g., in EUR, Figure 1A) does not necessarily contradict a higher burden of risk alleles in aggregate (Figure 2A, also see below), because the depleted allele at a locus may be non-risk or the global mean may be influenced by high frequencies in other groups. This suggests ancestry-specific architectures in which partially distinct collections of variants contribute to GBM susceptibility across lineages.

### 3.3. Composite Genetic Risk and Ancestry Gradients

Consistent with epidemiology and prior modeling results, the composite (unweighted) psPRS show the highest medians in EUR, intermediate values in SAS/AMR, and the lowest medians in EAS, with AFR tending toward higher values at these candidate loci. Such gradients are expected when risk alleles are more frequent in EUR and less frequent in EAS; this mirrors the RAF-based incidence modeling and ancestry risk trends in multiethnic cohorts. At the same time, diaspora and admixed subpopulations (e.g., AMR, ASW/ACB) manifest broad dispersion and “checkerboard” enrichment/depletion patterns, reflecting variable contributions of European, African, and Indigenous American ancestries; the intermediate placement of AMR in PCoA is concordant with this admixture profile.

The intermediate polygenic risk scores (PRS) observed in African populations, despite their low GBM incidence, likely reflect limitations of PRS portability combined with ancestry-specific genetic architecture. PRS derived from European GWAS capture only a fraction of inherited risk and may overestimate susceptibility in African populations due to linkage disequilibrium mismatch and omission of ancestry-specific protective variants. Moreover, the absence of major-effect loci, differences in somatic tumor evolution, and non-genetic modifiers likely attenuate the translation of germline risk into disease incidence.

### 3.4. Clinical and Research Applications

The marked population-level differentiation observed at GBM risk loci has important implications for genetic research and emerging translational applications. Although polygenic risk scores are not currently used for GBM screening, they are increasingly applied in cancer genetics for etiological inference, risk stratification, and cohort enrichment. Extensive evidence shows that PRS performance depends strongly on ancestry-specific allele frequencies, linkage disequilibrium structure, and effect-size estimates, limiting the transferability of models trained predominantly in European cohorts to diverse populations without explicit adjustment [29,30]. The presence of monomorphic or near-monomorphic risk loci in some populations, as observed here, further constrains the applicability of universal genetic risk models [30].

These findings also have direct implications for GWAS design and interpretation. Population differences in allele frequencies and linkage disequilibrium structure are known to influence association signals and can lead to reduced power or biased inference if ancestry is not properly accounted for [27,29,30]. Trans-ancestry GWAS have therefore been increasingly advocated as a means of improving fine mapping and identifying causal variants, particularly at loci with strong population stratification [27,28]. GBM risk loci such as *CCDC26* (8q24.21) and *CDKN2B-AS1* (9p21.3), which show pronounced ancestry-dependent frequency differences, have well-established functional relevance in gliomagenesis and exemplify how population structure can shape both genetic associations and biological interpretation [13,34,39].

From a clinical research perspective, ancestry-dependent genetic risk architecture may influence the distribution of molecular GBM subtypes and should be considered in the design and interpretation of molecularly stratified epidemiological studies and clinical trials. Prior studies have demonstrated that estimated European genetic ancestry correlates with glioma and GBM incidence in admixed populations, supporting a measurable contribution of inherited genetic background to disease risk [38,39]. Failure to consider such structure may therefore confound comparisons across populations and obscure gene–environment interactions.

Future research should prioritize the inclusion of large, ancestrally diverse cohorts and the development of ancestry-aware or trans-ancestry genetic models. Integrative approaches combining germline population genetics with somatic tumor genomics and other omics layers are particularly promising for clarifying how inherited variation interacts with tumor evolution and molecular phenotype [6]. Expanding diversity in GBM genetics research is essential not only for improved biological understanding but also to ensure equitable translation of genetic findings and to avoid perpetuating Eurocentric bias in neuro-oncology research [29,30].

### 3.5. Methodological Considerations and Limitations

First, the marker panel is disease-enriched and not representative of genome-wide polymorphism; therefore, conclusions pertain to GBM candidate loci rather than general diversity. Furthermore, the present study does not account for rare variants, structural variation, or gene-environment interactions, which most likely contribute to the reported disparities. Second, composite scores were unweighted, which avoids portability issues arising from ancestry-specific effect sizes but treats all variants equally, thereby underestimating the contribution of high-effect variants while overemphasizing common, low-effect alleles (see above). Third, while HWE departures were few, residual population structure within sampling frames and local LD patterns could still influence allele frequency and enrichment metrics. Fourth, although *Φ*_PT_ values were small, significance is driven by the large number of loci; effect sizes should be interpreted alongside distance metrics and PCoA rather than *p*-values alone. Fifth, the analyzed subpopulations did not uniformly represent the continental superpopulations. Finally, some risk alleles are absent in certain groups; downstream analyses that rely on per-locus effects (e.g., weighted PRS) must handle zero-frequency variants and LD proxies carefully.

## 4. Materials and Methods

### 4.1. Genetic and Population Data

Genome-wide significant candidate SNPs for GBM were extracted from the GWAS catalog (https://www.ebi.ac.uk/gwas/, accessed on 5 September 2025) using the search term “glioblastoma multiforme”. The genotypes were retrieved from the 1000 Genomes Phase 3 Project database [42] at https://ftp.1000genomes.ebi.ac.uk/vol1/ftp/release/20130502/ (accessed on 5 September 2025). Loci with missing genotypes were excluded to avoid an analysis bias. Finally, 673 loci were used for analysis (Table S1). The analysis was conducted at two levels of population structure: first, five major ethnic superpopulations were analyzed, followed by the analysis of 26 subpopulations (Table 3).

### 4.2. Analysis of Population Genetic Structure and Interpopulation Differentiation

All loci were checked for Hardy–Weinberg equilibrium (HWE). Genetic structure of the populations was estimated using standard parameters: allele frequencies, observed (*H*_o_) and expected (*H*_e_) heterozygosity [43], Wright’s fixation index (*F*, inbreeding coefficient) [44]. Interpopulation differentiation was estimated by *Φ*_PT_ from AMOVA [45,46], Nei’s genetic distances [47], and the log-likelihood ratio (*G*) based exact test [23]. Principal Coordinate Analysis was conducted using a pairwise *Φ*_PT_ matrix. The above analyses were performed using GenAlEx 6.5 [46] and GENEPOP v. 4.8.3 [48].

Enrichment and depletion patterns of alleles across populations were evaluated using a hypergeometric testing framework applied to allele count data. *p*-values were adjusted for multiple comparisons using the false discovery rate (FDR) method, and statistical significance was defined at an FDR threshold of 0.05. The adjusted *p*-values were transformed into signed −log_10_ scores to represent both the magnitude and direction of statistical significance, and these values were visualized using a heatmap to highlight patterns of allele enrichment and depletion across populations. For visualization purposes, values were capped at ±5 to avoid dominance of extreme signals. Both populations and alleles were clustered according to their average correlation structure.

### 4.3. Computation of Polygenic Risk Scores

The individual composite polygenic risk score (PRS) for GBM was computed following the approach described by Choi et al. and implemented in the PRSice-2 v. 2.3.5 software [49]:

(1) $$PRS=\sum_{i=1}^{N}\left(\beta_{i}\times {SNP}_{i}\right)$$

where *β*_i_ denotes the effect size of the *i*-th SNP obtained from the GWAS reference dataset, *SNP_i_* is the allele dosage (0, 1, or 2 under an additive model) for that SNP in the target individual, and *N* is the total number of SNPs shared between the base and target datasets.

Based on this, an individual carrying two copies of the risk allele at every locus attains a maximum score of 1, while an individual carrying no risk alleles has a score of 0. Because effect-size estimates were available only for European cohorts and are not easily generalizable to other ancestral groups [29], and the PRS was not weighted by these effect sizes. Nevertheless, we assumed that most GWAS-derived associations are broadly replicable across non-European populations [30]. Population-specific PRS (psPRS) values were calculated as the mean PRS across individuals within each population. Due to limited data about effect alleles for GBM risk loci, only 18 loci were used for PRS calculation.

To assess differences in polygenic risk scores across populations, a non-parametric statistical approach was used due to potential deviations from normality in the score distributions. First, the Kruskal–Wallis rank-sum test was performed to estimate whether significant differences existed among the groups. This test assesses whether the distributions of the variable of interest differ across multiple independent groups without assuming normality. If the Kruskal–Wallis test indicated a statistically significant result (*p* < 0.05), post hoc pairwise comparisons were conducted using Dunn’s test to identify which specific groups differed from each other. To control for multiple testing, *p*-values obtained from Dunn’s test were adjusted using the Benjamini–Hochberg false discovery rate (FDR) correction. All statistical analyses were performed in the R environment using the FSA package, v. 0.10.1 [50].

## 5. Conclusions

The present study provides compelling evidence that major ethnic populations are significantly differentiated at GBM risk loci. Our findings demonstrate that the genetic architecture underlying GBM susceptibility is not uniformly distributed worldwide but instead exhibits population stratification, which significantly contributes to the documented ethnic disparities in disease prevalence.

This has immediate implications for study design (trans-ancestry GWAS, fine mapping) and clinical translation: risk prediction models trained in EUR cohorts will have limited portability to EAS, AFR, and AMR populations unless they incorporate ancestry-specific allele frequencies, LD structure, and effect estimates. Furthermore, the absence or rarity of certain alleles in some populations suggests that alternative biological pathways (tagged by different variants) may underlie GBM susceptibility in those groups.

## Figures and Tables

**Table 1:** The parameters of genetic variation at the GBM risk loci for the ethnic superpopulations.

Populations	*H*_o_	*H*_e_	*I*	*F*
AFR	0.041 ± 0.004	0.042 ± 0.004	0.072 ± 0.006	0.004 ± 0.001
AMR	0.040 ± 0.003	0.041 ± 0.003	0.083 ± 0.005	0.008 ± 0.002
EAS	0.024 ± 0.003	0.025 ± 0.003	0.042 ± 0.005	0.015 ± 0.002
EUR	0.051 ± 0.003	0.051 ± 0.003	0.107 ± 0.005	0.006 ± 0.002
SAS	0.040 ± 0.003	0.041 ± 0.003	0.078 ± 0.005	0.009 ± 0.002

Note: *H*_o_—observed heterozygosity; *H*_e_—expected heterozygosity; *I*—Shannon’s information index; *F*—fixation index.

**Table 2:** A matrix of the pairwise *Φ*_PT_ values and Nei’s genetic distances between the ethnic superpopulations.

	AFR	AMR	EAS	EUR	SAS
AFR		0.004	0.006	0.006	0.005
AMR	0.167		0.002	0.001	0.001
EAS	0.260	0.096		0.003	0.002
EUR	0.193	0.030	0.147		0.001
SAS	0.177	0.050	0.119	0.052	

Note: *Φ*_PT_ values are given below the diagonal, Nei’s genetic distances are given above the diagonal. All *Φ*_PT_ values are significant at *p* ≤ 0.001.

**Table 4:** The *p*-values of the psPRS pairwise comparisons across superpopulations using Dunn’s test with Benjamini–Hochberg correction.

Population Pair	Z	*P*unadj	*P*adj	Significance
AFR—AMR	5.275	1.3 × 10^−7^	2.2 × 10^−7^	***
AFR—EAS	18.140	1.5 × 10^−73^	7.7 × 10^−73^	***
AMR—EAS	10.365	3.6 × 10^−25^	9.0 × 10^−25^	***
AFR—EUR	−2.847	0.0044	0.0049	**
AMR—EUR	−7.425	1.1 × 10^−13^	2.3 × 10^−13^	***
EAS—EUR	−19.693	2.5 × 10^−86^	2.5 × 10^−85^	***
AFR—SAS	2.115	0.0344	0.0344	*
AMR—SAS	−3.185	0.0014	0.0018	**
EAS—SAS	−14.912	2.7 × 10^−50^	9.2 × 10^−50^	***
EUR—SAS	4.639	3.5 × 10^−6^	5.0 × 10^−6^	***

Note: Results of pairwise comparisons of the genetic risk score between populations based on Dunn’s post hoc test following a significant Kruskal–Wallis test. *p*-values were adjusted for multiple testing using the Benjamini–Hochberg (FDR) procedure. The table reports the raw (*P*unadj) and adjusted (*P*adj) *p*-values along with corresponding significance levels (*** *p* < 0.001, ** *p* < 0.01, * *p* < 0.05).

## Data Availability

No new data were created or analyzed in this study. The original individual genotype data for each analyzed locus are freely available from the 1000 Genomes Project at https://ftp.1000genomes.ebi.ac.uk/vol1/ftp/release/20130502/ (accessed on 5 September 2025).

## Supplementary Materials

The following supporting information can be downloaded at: https://www.mdpi.com/article/10.3390/ijms27104424/s1.

## Abbreviations

AFR	African (superpopulation)
AMR	Admixed American (superpopulation)
AMOVA	Analysis of Molecular Variance
*CCDC26*	Coiled-Coil Domain Containing 26
*CDKN2A*	Cyclin-Dependent Kinase Inhibitor 2A
*CDKN2B*	Cyclin-Dependent Kinase Inhibitor 2B
*CDKN2B-AS1*	CDKN2B Antisense RNA 1
EAS	East Asian (superpopulation)
*EGFR*	Epidermal Growth Factor Receptor
EUR	European (superpopulation)
FDR	False Discovery Rate
FIN	Finnish (subpopulation)
GBM	Glioblastoma Multiforme
GWAS	Genome-Wide Association Study
HWE	Hardy–Weinberg Equilibrium
*IDH1*	Isocitrate Dehydrogenase 1
*IDH2*	Isocitrate Dehydrogenase 2
PCoA	Principal Coordinate Analysis
PRS	Polygenic Risk Score
psPRS	Population-Specific Polygenic Risk Score
*PTEN*	Phosphatase and Tensin Homolog
RAF	Risk Allele Frequency
SAS	South Asian (superpopulation)
SNP	Single Nucleotide Polymorphism
*TERT*	Telomerase Reverse Transcriptase
*TP53*	Tumor Protein 53
